# Supplementary material for: SCassist: An AI Based Workflow Assistant for Single-Cell Analysis
Source: bioRxiv. 2025 Apr 28:2025.04.22.650107. Preprint. [Version 2] doi: 10.1101/2025.04.22.650107 (PMC12148057; doi:10.1101/2025.04.22.650107)
Supplement: Supplement 4 [file media-4.pdf]

Supplementary Data in this File

Use of Large Language Models:

Authors declare that Google Gemini and Meta's Llama3 were used as an aid to correct written text, as an algorithmic technique for research study, as an evaluation technique to identify anomalies in our code and text, as a code writing assistant and for document creation. The authors have reviewed any content generated by Gemini and Llama3.

This supplementary file includes the following content:

Supplementary Table 3: Publicly available single-cell datasets used for SCassist evaluation.
Supplementary Table 4: Template scoring sheet (Likert scale) for Expert Human Evaluation.
Supplementary Table 5: Ground truth categories and Groundedness score.
Supplementary Table 6: BERT based semantic similarity scores for SCassist generated reports.
Supplementary Figure 1: Frequency Tables and Frequency Distribution of expert human evaluation scores.
Supplementary Table 7: Summary statistics on human expert evaluation, using the Likert Scale of 1 to 5. 1 being worst performing, 3 average performing and 5 best performing
Supplementary Table 8: The Wilcoxon Rank-Sum test showing no statistically significant differences in the LLM's performance across datasets and expert levels.

Supplementary Table 3: Publicly available single-cell datasets used for SCassist evaluation.

Pubmed ID	Availability	Sample Type	Total No. of Cells
39085199	GitHub Link filtered_feature_bc_matrix.h5	PBMCs from Birdshot Uveitis Patient	14300
39085199	GitHub Link filtered_feature_bc_matrix.h5	PBMCs from Healthy Human Control	24790
38697116	NCBI GEO GSM6625298 Link filtered_feature_bc_matrix.h5	NK, CD4+ and CD8+ T cells from LCMV infected WT mice	5666
38697116	NCBI GEO GSM6625299 Link filtered_feature_bc_matrix.h5	NK, CD4+ and CD8+ T cells from LCMV infected Ifng - CTCF binding site mutant mice	4885

Supplementary Table 4: Template scoring sheet (Likert scale) for Expert Human Evaluation.

Score	Accuracy
1	The results and recommendations are completely inaccurate
2	The results and recommendations are slightly inaccurate
3	The results and recommendations are acceptable
4	The results and recommendations are mostly accurate
5	The results and recommendations are highly accurate
	**Relevance**
1	The results and recommendations are not relevant to this study at all
2	The results and recommendations are slightly relevant to this study
3	The results and recommendations are relevant to this study
4	The results and recommendations are mostly relevant to this study
5	The results and recommendations are highly relevant to this study
	**Clarity**
1	The results and recommendations are very difficult to understand
2	The results and recommendations are difficult to understand
3	The results and recommendations are clear
4	The results and recommendations are easy to understand
5	The results and recommendations are very easy to understand
	**Trustworthiness**
1	Doesn't make sense, so doesn't seem trustable at all
2	Doesn't make much sense, so doesn't seem very trustable
3	Makes some sense, so seems somewhat trustable
4	Makes sense, so seems trustable
5	Makes perfect sense, so seems completely trustable
	**Overall Satisfaction**
1	Completely useless, so would not recommend at all
2	Somewhat useless, would not recommend to most
3	Somewhat useful, would recommend with reservations
4	Useful, would recommend
5	Very useful, would highly recommend

Supplementary Table 5: Ground truth categories and Groundedness score

	Input Tokens	**SCassist-LCMV-Grounded**	Total reported	Groundedness score
Data Metrics	16	14	14	100
Variable Genes	30	22	22	100
Variance Explained	10	10	11	90.91
PC Genes	250	73	73	100
Annotation Genes	630	234	234	100
Enriched Terms	3176	53	53	100
Network Genes	24	23	23	100
**Average**				**98.70**
**Total**	**4136**			

	Input Tokens	**SCassist-BCRUV-Grounded**	Total reported	Groundedness score
Data Metrics	16	14	14	100
Variable Genes	30	29	29	100
Variance Explained	10	1	1	100
PC Genes	250	67	67	100
Annotation Genes	450	182	183	99.45
Enriched Terms	3327	28	28	100
Network Genes	27	25	25	100
**Average**				**99.92**
**Total**	**4110**			

Supplementary Table 6: BERT based semantic similarity scores for SCassist generated reports

	Input Tokens	**SCassist-LCMV-Similarity Score**
KEGG_pathways report	99	0.76059038
GO_terms report	2196	0.73808815
Network summary	24	0.79054358
**Total/Average**	**2319**	**0.763074037**

	Input Tokens	**SCassist-BCRUV-Similarity Score**
KEGG_pathways report	117	0.7475318
GO_terms report	436	0.7515198
Network summary	27	0.7080501
**Total/Average**	**580**	**0.735700567**

Supplementary Figure 1: Frequency Tables and Frequency Distribution of expert human evaluation scores.

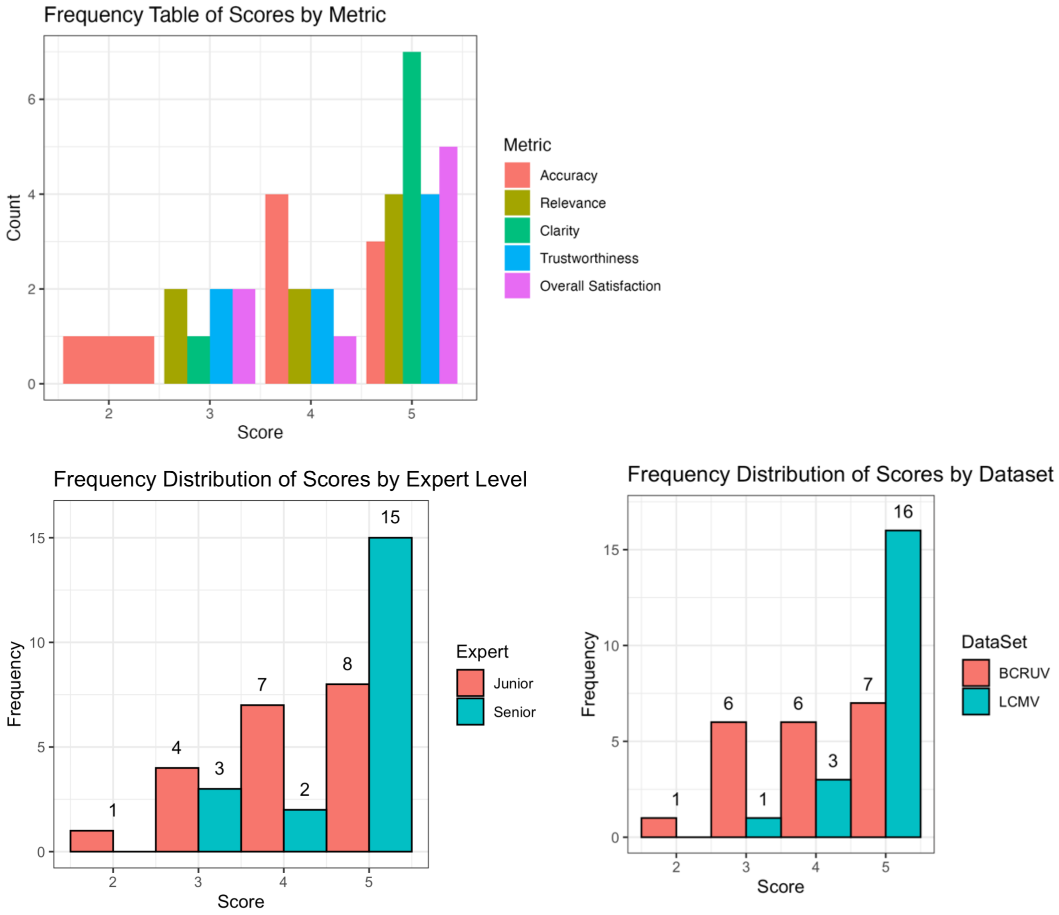

Supplementary Table 7: Summary statistics on human expert evaluation, using the Likert Scale of 1 to 5. 1 being worst performing, 3 average performing and 5 best performing

**Metric**	**Mean**	**Median**	**Min**	**Max**	**StdDev**
**Accuracy**	4.125	4	2	5	0.99
**Relevance**	4.25	4.5	3	5	0.89
**Clarity**	4.75	5	3	5	0.71
**Trustworthiness**	4.25	4.5	3	5	0.89
**Overall Satisfaction**	4.375	5	3	5	0.92

Supplementary Table 8: The Wilcoxon Rank-Sum test showing no statistically significant differences in the LLM's performance across datasets and expert levels.

**BCRV vs LCMV**	
Metric	p_value
Accuracy	1
Relevance	1
Clarity	1
Trustworthiness	0.80
Overall Satisfaction	0.33

**Seniors vs Juniors**	
Metric	p_value
Accuracy	0.29
Relevance	1
Clarity	1
Trustworthiness	1
Overall Satisfaction	1
